# Pacing, Exercise Intensity, and Technique by Performance Level in Long-Distance Cross-Country Skiing

**DOI:** 10.3389/fphys.2020.00017

**Published:** 2020-02-14

**Authors:** Thomas L. Stöggl, Markus Hertlein, Richard Brunauer, Boye Welde, Erik P. Andersson, Mikael Swarén

**Affiliations:** ^1^Department of Sport and Exercise Science, University of Salzburg, Salzburg, Austria; ^2^Red Bull Athlete Performance Center, Salzburg, Austria; ^3^Salzburg Research Forschungsgesellschaft m.b.H., Salzburg, Austria; ^4^School of Sport Sciences, UiT The Arctic University of Norway, Tromsø, Norway; ^5^Swedish Winter Sports Research Centre, Department of Health Sciences, Mid Sweden University, Östersund, Sweden; ^6^Swedish Olympic Academy, Stockholm, Sweden

**Keywords:** competition, global navigation satellite system, heart rate, physiological load, racing, skiing speed, technique application, Vasaloppet

## Abstract

**Introduction:**

Long-distance cross-country skiing (XCS) has gained increased popularity within the past decades. However, research about long-distance XCS is limited; therefore, the aim of this study was to analyze the intensity distribution, technique application, and pacing strategies during long-distance XCS racing.

**Methods:**

Heart rate (HR) and section skiing speeds of 9 elite (ranked 1–100) and 10 amateur skiers (ranked 101–1,500) during the 90-km Vasaloppet race were collected. In addition, during the first uphill, the first 1,000 skiers were video-recorded to analyze the applied skiing strategy (e.g. grip-waxed skis versus exclusive double poling).

**Results:**

Mean race intensity was 82% of maximal HR and was not different between performance groups even though elite skiers skied ∼15% faster than amateurs. There was an interaction effect of section × group with a pronounced decrease in HR in amateurs compared with more even pacing in elite skiers (0.13 vs. 0.04% decrease/km) and skiing at higher percentage in the high-intensity zones in elite compared with amateurs (46 vs. 24%). Ninety-eight percent of the top 100 skiers and 59% of the first 1,000 skiers used exclusively double poling.

**Conclusion:**

Elite and amateur skiers ski at comparable mean race exercise intensity, but they have clear differences in skiing speed. The difference in the pacing profiles between elite and amateur skiers (more even vs. distinct positive pacing) demonstrate the greater capacity of the former with respect to physiological capacity and highlights that amateurs seem to start too fast according to their capacities. The exclusive application of the double poling technique is no longer a phenomenon of elite skiers but is widely used among the top 1,000 ranked skiers.

## Introduction

Cross-country skiing (XCS) is one of the most demanding endurance sports and includes Olympic competition distances from approximately 1–50 km (Stöggl et al., 2018). Besides the competition series hosted by the Fédération International de Ski (FIS) (e.g. World Cup, European Cup, FIS races), also a great number of long-distance popular races exist. The skiing distances are usually from half marathon up to 220 km and were originally addressed to recreational and amateur skiers. However, special pro-series like the “Ski-Classics” and “Wordloppet” have recently emerged, leading to an increased professionalization of participants (e.g. more pro-teams, higher price money) (Sandbakk and Holmberg, 2017; Sagelv et al., 2018). Despite this popularity, research about long-distance XCS is sparse.

In long-distance events, winning margins are often mere fractions of a second, and tactics (e.g. drafting) and the pacing strategy may be race decisive. Of special focus for the current study is the research about pacing and the physiological load for race durations >3 h. In endurance events lasting more than 4 h, the exercise intensity has been shown to progressively decrease (positive pacing) (e.g. O’Toole et al., 1998; Laursen et al., 2002; Neumayr et al., 2002, 2004; Lambert et al., 2004; Abbiss et al., 2006). Furthermore, the fastest marathon and 100 km ultra-marathon runners (Lambert et al., 2004; Hanley, 2016) demonstrated a more even-paced running, whereas slower athletes showed more of a decreasing speed across the race (i.e. more of a positive pacing profile).

In connection with XCS races, the undulating terrain, effects of equipment (e.g. glide wax, grip wax, ski preparation, ski properties) and external conditions (air, snow, humidity, radiation, dirt, etc.) constitute a situation more complex than in other endurance sports (Stöggl et al., 2018). As recently summarized by Stöggl et al. (2018), the majority of studies about XCS demonstrated that, in general, skiers apply a positive pacing strategy irrespective of distance (5–90 km), technique, or sex with higher-performing athletes using a more even pacing strategy as compared with the lower performing and/or less experienced athletes.

However, there is sparsity with respect to research on the physiological response during prolonged racing in different sports. Formenti et al. (2015) stated that the pure analysis of the pacing strategy (speed or power output as external load) is not sufficient. This information needs to be evaluated in relation to the exercise intensity based on the physiological response (internal load), as for instance using heart rate (HR) measures (e.g. Gilman, 1996). While St Clair Gibson et al. (2001) demonstrated a significant increase in HR of ∼8% during a 100-km cycling time trial (∼2.5 h), HR was shown to decline at an average of 1–2%/h during cycling and triathlon events lasting 6–25 h (Neumayr et al., 2002, 2004; Laursen et al., 2005) and 6–7% for prolonged running or cycling (>6 h) (O’Toole et al., 1998). Only the study of Bilodeau et al. (1996) focused on the physiological load during long-distance racing (30 and 50 km). In their study, no changes in physical exertion (based on HR) were found. It is worth noting that potential associations between race performance and the alterations in HR or the distribution of intensity across the races were not yet examined in any of these studies. Furthermore, especially in classical style long-distance races, the distinct development of the double poling (DP) technique (i.e. exclusive application of DP across the entire race), might have altered aspects about pacing and the physiological load during an XCS race compared with the previous studies. To note, that the amount of skiers applying the strategy of exclusive DP during a long-distance race, and if this phenomenon is restricted to elite skiers, was not yet scope of any study. Therefore, the question remains if faster skiers are able to ski at a lower relative exercise intensity [e.g. at a lower% of maximal HR (HR_max_)] at the first half of the race and reach higher relative intensities at the second half of the race (e.g. less pronounced positive or more even pacing with respect to HR) compared with lower performing skiers.

The aims of the current study were to describe (1) the physiological load (as HR distribution in different intensity zones), (2) the pacing strategy with respect to section times and HR response, and (3) the ski-style application (i.e. grip-waxed skis or not grip-waxed skis using exclusively DP) between elite (top 100) versus recreational to amateur level participants (rank 101–1,500). The specific hypotheses are that higher ranked skiers demonstrate more even pacing and lower exercise intensity during the first half of the race but higher load during the second part and use, to a greater extent, the strategy of exclusive DP, while lower ranked skiers show the opposite.

## Materials and Methods

### The Vasaloppet Race

**FIGURE 1:**
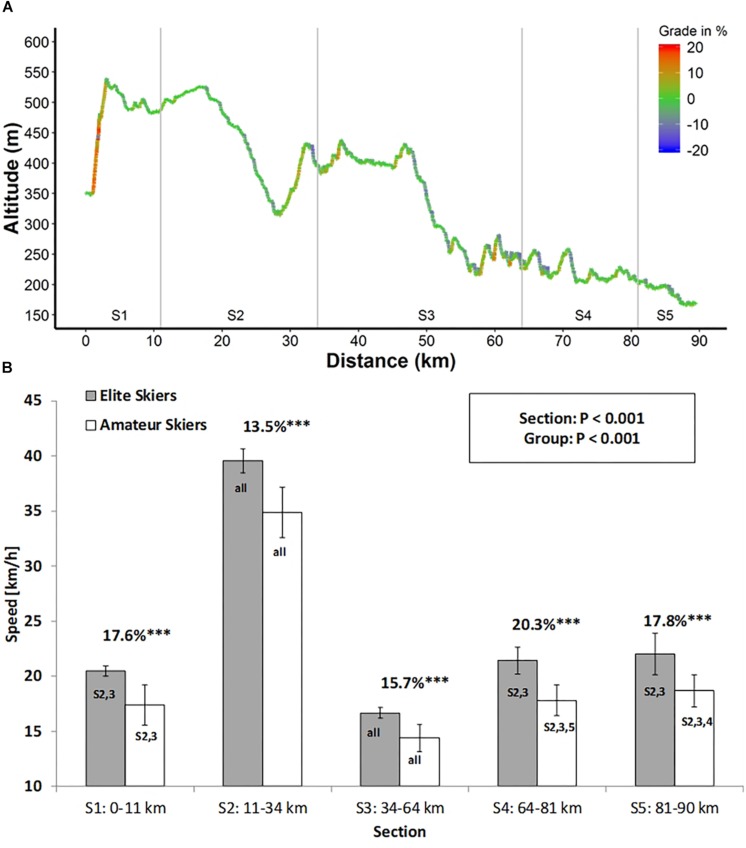
**(A)** Track profile of the 90-km Vasaloppet and **(B)** section speeds for both groups and percent difference between groups in mean speed; S1, S2,…, S5, section 1, section 2,…, section 5; ***P < 0.001, significantly different to amateur skiers (mean ±SD). S1, 2,.…,5, significantly different to the respective section within each group.

The “Vasaloppet” is one of the oldest and longest XCS race in the world and has the largest rates of participation. In 2017, 15,800 participants signed in for the race, which covers a distance of 90 km. Since 1922, when the first Vasaloppet was held, it starts in Sälen and finishes in Mora. In between the start and finish, there are seven checkpoints and feeding stations. The course consists of various flat sections and sections with slight undulating terrain and with two noteworthy hills, with the first directly located after the start and the second after at ∼28 km (see Figure 1A). The record time is 3:38:41 (h:min:s) from the year 2012, but the winning time varies due to different variables such as snow conditions, weather, and/or tactics. Based on the average skiing performance over the last 20 years, the first male participant crosses the finish line after around 4 h, while the last participants that make the cut-off need up to 14 h. The race was first time won with exclusive application of DP (glide wax across the entire ski base without using grip wax) in the year 2013. Following this, all victories in the past years were won with pure DP, and a trend toward an increase in the total number of “pure double polers” can be observed, while this aspect was not yet systematically analyzed.

### Participants and Data Input

Before and after the 90-km Vasaloppet 2017, instructions about the study and a questionnaire were sent via email to all Ski Classics team captains, the Vasaloppet organization team, and various Swedish, Finnish, Austrian, German, and Norwegian XCS clubs and athletes to collect information about personal and race-specific aspects. Participants were fully informed about the study details and participation requirements with written information before providing written informed consent to participate. The study received approval from the local Ethical Committee (EK-GZ: 05/2017) and was conducted in accordance with the Declaration of Helsinki.

The questionnaire consisted of the following items: body weight, body height; pole length (from tip to strap according to FIS rules); final ranking in the race; HR_max_ from a recent (last 5 months before the race) time to exhaustion ramp protocol in the laboratory or based on a field test with running or roller skiing; application of grip wax or pure DP during the race; and qualitative information about the glide and grip conditions, tactics, intended pacing strategy, and weak and strong parts during the race. In addition, if they used an HR monitor during the race, they were asked to send the raw data [HR and Global Navigation Satellite System (GNSS) data]. Twenty-eight participants responded and provided their data from the race. Two skiers did not use HR belts, so only the GNSS data of their watches were available. For three other skiers, the HR data were not applicable based on too many artifacts and missing values in the data. Two skiers were excluded based on the too low race performance (ranked between 4,000 and 5,500) compared with the other skiers included. Only two women have sent their HR data. Therefore, to keep the sample homogeneous with respect to biological sex, their data were not included in the analysis. The final sample for statistical analysis consisted of 19 male skiers (body height, 182 ± 8 cm; body weight, 78 ± 8 kg) ranked from 1st to 1,337th place. The skiers were divided into elite skiers (rank 1–100, *n* = 9), and amateur and recreational level skiers (rank 101–1,337, *n* = 10). On the race day, the tracks were freshly groomed and hard packed. The day before the race, there was distinct snow fall leading to a mix of old and new snow conditions. The air temperature was around −7°C in the morning at the start (Sälen) and around −3°C when the winner reached the finish line at Mora. The elite had a headwind of approximately 4–8 m/s all the way (information provided by the race organizer).

### Data Processing

The course was divided into five sections (S1, start to Smågan, 0–11 km; S2, Smågan to Mångsbodarna, 11–34 km; S3, Mångsbodarna to Oxberg, 34–64 km; S4, Oxberg to Eldris, 64–81 km; and S5, Eldris to finish, 81–90 km). Based on the official result list with all split times and the official section distances^1^ together with the raw files for HR of the athletes, the intensity distribution and mean section/race speed were calculated. Based on the GNSS raw data of the HR monitors, the covered distance and total vertical climb for the entire race and each single section were calculated. The HR data were converted into relative values based on HR_max_ values provided by the questionnaire data of the skiers. An edited version of the five-intensity zone model by Tonnessen et al. (2014) was used (zone 1, 55–71%; zone 2, 72–81%; zone 3, 82–86%; zone 4, 87–91%; and zone 5, 92–100% of HR_max_). In addition, the intensity zones 1–3 were pooled as “low” (55–86%) and zones 4 and 5 as “high” (87–100%) intensity zones (binary model).

### Technique Analysis With 2D Video

In addition to the questionnaire data about the applied ski technique strategy (grip wax or exclusive DP), a video analysis was performed at the mid part of the first long uphill (at ∼2.5 km), and the first 1,000 skiers passing this checkpoint were filmed. The video camera (Sony HDR-PJ810E, Sony corp., Tokyo, Japan) was positioned perpendicular to the track 1.5 m above the ground on top of a tripod and was set at 50 Hz with a shutter speed of 1/500 s and recorded the skiers at high resolution (1,920 × 1,080 progressive scan). An XCS expert analyzed two times the video data and recorded if a skier used the diagonal stride and/or kick DP (using grip wax or other climbing aids) or exclusively the DP technique (assuming no grip wax and exclusive DP across the full 90-km race).

### Statistics

All results are presented as means ±SD. Variability within selected variables was calculated by the coefficient of variation (CV) = SD/mean × 100 (%). To compare mean skiing speed (based on section times) and HR response for each intensity zone (zones 1–5 and low vs. high) within the five race sections, two-way 2 × 5 ANOVAs with repeated measures (five sections) and performance group (elite vs. amateur) were applied. *Post hoc* comparisons with Bonferroni corrections were conducted to detect differences. In those instances where the sphericity assumption was violated, *P*-values were adjusted according to the Huynh–Feldt correction when epsilon was >0.75 and with the Greenhouse–Geisser correction when epsilon was <0.75. The effect size was evaluated as _*p*_η^2^ (partial eta squared) (with 0.01 < _*p*_η^2^ < 0.06 considered to be a small, 0.06 < _*p*_η^2^ < 0.14 a medium, and _*p*_η^2^ > 0.14 a large effect) or for the *t*-tests as Cohen’s *d* (0 < *d* < 0.2 considered to be a very small, 0.2 < *d* < 0.5 a small, 0.5 < *d* < 0.8 a medium, and *d* > 0.8 a large effect). For all analyses, the level of statistical significance was set at α = 0.05. All statistical analyses were carried out utilizing the SPSS 24.0 (SPSS Inc., Chicago, IL, United States), and graphs were done using R-Studio Version 1.1.453 (R version 3.5.1) and Office Excel 2016 (Microsoft Corporation, Redmond, WA, United States).

## Results

### Overall Race Performance and Intensity

During the 2017 race, the skiing speed was quite high from the beginning, and a group of 29 skiers was able to leave the followers for the first few checkpoints and has grown again to 55 skiers until Hökberg. Mean race time for the entire group was 4:30:55 ± 0:28:14 (h:min:s) (elite, 4:06:01 ± 0:08:55; amateurs: 4:49:02 ± 0:22:52) corresponding to a mean skiing speed of 20.1 ± 2.0 km/h with the elite athletes skiing 15% faster compared with the amateur level skiers (22.0 ± 0.8 km/h vs. 18.8 ± 1.4 km/h, *P* < 0.001). Mean HR across the entire race was 82 ± 4% of HR_max_ for the total group and was higher in elite versus amateur skiers with respect to absolute values (161 ± 9 bpm vs. 151 ± 10 bpm, *P* = 0.043) but with no difference in relative HR (83 ± 5% vs. 81 ± 3% of HR_max_, *P* = 0.154). Peak HR was 96 ± 5% of HR_max_ in the total group and was similar between the groups (*P* = 0.323). Peak HR was detected in the first long uphill after the start (km 1–3) in 17 skiers and in the final spurt for two skiers (the two fastest in the group).

### Performance and Intensity Within Sections

All five sections were different to each other with respect to mean skiing speed (main effect section: *P* < 0.001), with highest speeds in section 2 and lowest in section 3. Section speed was higher for the elite athletes compared with the amateur skiers for all sections (main effect group, *P* < 0.001). There was an interaction effect of section × group (*P* = 0.002) with greatest relative speed differences between the two groups in section 4 (Oxberg to Eldris) and lowest in sections 2 and 3 (Figure 1B). Greatest variability within a section was for the amateurs in section 1 [coefficient of variation (CV) = 11%] with constant CVs of 7–8% in the remaining four sections, while for the elite, the highest variability was found in the final section (CV = 9%) with increasing variability from section 1 to 5 (CVs, 2, 3, 3, 6, and 9%).

Based on the individual GNSS data provided by the athletes, the 90-km course was 90.4 ± 0.3 km (range, 87.93–91.12 km, CV = 0.3%), and the mean total climb was 1,146 ± 254 m (range, 616–1,445 m, CV = 22%). For the total distance, this represents a bias of 192 m (0.21%) and a limit of agreement of 806 m (0.90%), and for the total climb, a bias of -234 m (17%) with a limit of agreement of 436 m (30%). The official values should be exactly 90-km distance and 1,380 m total climb.

### Exercise Intensity Distribution During the Race Based on HR

**FIGURE 2:**
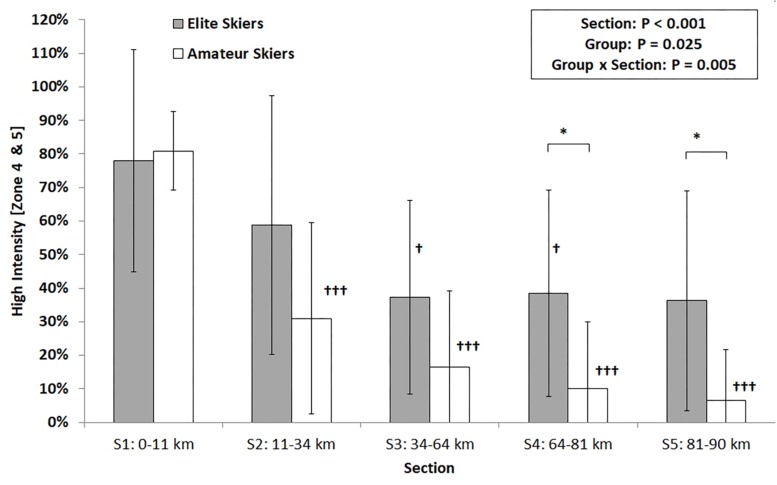
Proportion of high intensity (zones 4 and 5) within the five sections of the 90-km Vasaloppet race for elite and amateur skiers. *P < 0.05, significantly different to amateur skiers; ^†^, ^†^^†^^†^*P* < 0.05, *P* < 0.001, significantly different to section 1.

The exercise intensity distribution during the 90-km race for the entire group was 6% in zone 1, 32% in zone 2, 29% in zone 3, 23% in zone 4, and 11% in zone 5. Over the total race distance, there was a decrease in high intensity (main effect section, *P* < 0.001) and a main effect of group with elite athletes demonstrating a higher percentage of high intensity compared with amateur level skiers (46 ± 26% vs. 24 ± 17%, *P* = 0.025). Furthermore, an interaction effect of section × group with respect to high intensity was found (*P* = 0.005). In amateur skiers, the percentage within high was highest in section 1, with lower values in the remaining sections (sections 2–4 <section 1, all *P* < 0.001), while only a trend (main effect section, *P* = 0.097) was found in elite skiers with significantly lower exercise intensity in section 3 and 4 in comparison with section 1 (*P* = 0.011; *P* = 0.041) (Figure 2).

**FIGURE 3:**
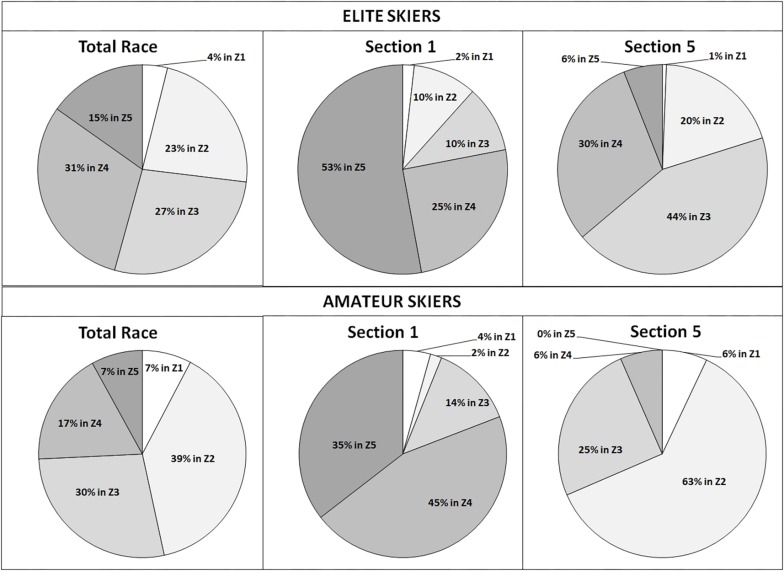
Percent distribution of exercise intensity within the five intensity zones (zone 1, 55–72%; zone 2, 72–82%; zone 3, 82–87%; zone 4, 87–92%; and zone 5, 92–100% of HR_max_) for elite skiers and amateur skiers over the total 90-km race, the first section (section 1, 0–11 km) and last section (section 5, 81–90 km).

The detailed distribution of the exercise intensity between elite and amateur level athletes with respect to all five intensity zones for the total race, the starting section (1–11 km), and the finish section (81–90 km) are presented in Table 1 and Figure 3.

**TABLE 1:** Mean skiing speeds and intensity distribution (five zones based on heart rate values in% of maximal heart rate) within the five sections (S1 to S5) of the 90-km Vasaloppet race for elite skiers (Ranked top 100, *n* = 8) and amateur level skiers (ranked from 101 to 1700, *n* = 11).

	**Group**	**S1**	**S2**	**S3**	**S4**	**S5**	**Total**	**ANOVA**
Mean speed (km/h)	Elite	20.5±0.5	39.6±1.1	16.7±5	21.4±1.2	22.0±1.9	22.0±8	^a^*F*_4,14_ = 1703; *p* < 0.001; _*p*_η^2^ = 1.00; pow = 1.00
	Amateur	17.4±1.8***	34.9±2.2***	14.4±1.2***	17.8±1.4***	18.7±1.5***	18.78±1.4***	^b^*F*_1,17_ = 33; *p* < 0.001; _*p*_η^2^ = 0.66; pow = 1.00 ^c^*F*_4,14_ = 7.6; *p* = 0.002; _*p*_η^2^ = 0.68; pow = 0.97

%Zone 1	Elite	2±2	3±7	6±7	3±6	1±2	4±5	^a^*F*_4,14_ = 2.4; *p* = 0.095; _*p*_η^2^ = 0.41 ^b^*F*_1,17_ = 2.7;
	Amateur	4±6	2±3	7±7	12±13	7±9	7±6	*p* = 0.118; _*p*_η^2^ = 0.14 ^c^*F*_4,14_ = 1.8; *p* = 0.184; _*p*_η^2^ = 0.34

%Zone 2	Elite	10±21	20±28	29±20	24±21	19±23	23±19	^a^*F*_4,14_ = 16; *p* < 0.001; _*p*_η^2^ = 0.82; pow = 1.00
	Amateur	2±1	21±17	41±18	55±24*	62±30**	38±16	^b^*F*_1,17_ = 4.9; *p* = 0.042; _*p*_η^2^ = 0.22; pow = 0.55 ^c^*F*_4,14_ = 4.4; *p* = 0.017; _*p*_η^2^ = 0.56; pow = 0.82

%Zone 3	Elite	11±12	18±18	28±12	34±18	18±16	27±11	^a^*F*_4,14_ = 5.9; *p* = 0.005; _*p*_η^2^ = 0.63; pow = 0.92
	Amateur	13±10	46±24*	36±17	23±19	48±23**	30±11	^b^*F*_1,17_ = 9.9; *p* = 0.006; _*p*_η^2^ = 0.37; pow = 0.84 ^c^*F*_4,14_ = 3.4; *p* = 0.038; η^2^ = 0.49; pow = 0.71

%Zone 4	Elite	25±20	41±29	27±15	34±25	30±26	31±13	^a^*F*_4,14_ = 2.9; *p* = 0.063; _*p*_η^2^ = 0.45 ^b^*F*_1,17_ = 8.2;
	Amateur	45±22*	24±16	12±11*	9±16*	7±15*	17±8*	*p* = 0.011; _*p*_η^2^ = 0.33; pow = 0.77 ^c^*F*_4,14_ = 4.2; *p* = 0.020; _*p*_η^2^ = 0.55; pow = 0.80

%Zone 5	Elite	53±33	18±24	10±19	5±6	6±12	15±15	^a^*F*_4,14_ = 11; *p* < 0.001; _*p*_η^2^ = 0.76; pow = 1.00
	Amateur	36±29	7±18	4±13	1±4	0±0	7±11	^b^*F*_1,17_ = 2.4; *p* = 0.139; _*p*_η^2^ = 0.12 ^c^*F*_4,14_ = 0.9; *p* = 0.511; _*p*_η^2^ = 0.59

S1, Start (km 0) to Smågan (km 11); S2, Smågan (km 11) to Mångsbodarna (km 34); S3, Mångsbodarna (km 34) to Oxberg (km 62); S4, Oxberg (km 62) to Eldris (km 81); S5, Eldris (km 81) to Finish (km 90). ^*a*^Main effect of section. ^*b*^Main effect of performance group. ^*c*^Interaction effect section × performance group. *P < 05; **p < 01; ***p < 001, significantly different to Elite; _*p*_η^2^, partial eta squared effect size; pow, statistical power.

**FIGURE 4:**
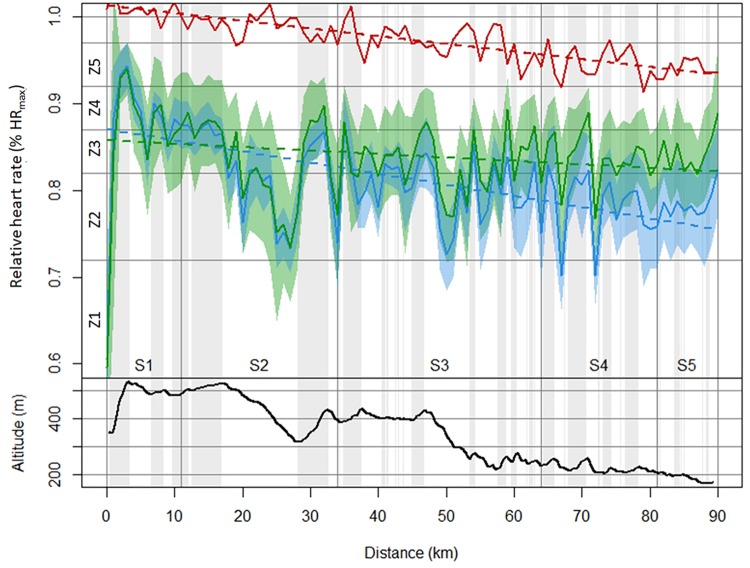
Relative exercise intensity of elite (green) and amateur level (blue) skiers across the 90-km Vasaloppet race. The black line represents the altitude profile with gray-shaded areas illustrating uphill sections; the red line represents the difference in the mean exercise intensity between the groups, with the scattered red line representing the linear regression.

Figure 4 illustrates the course (calculation is based on 10-m steps, visualization in 1,000-m steps) of the relative exercise intensity (% HR_max_) for both groups and the difference in the group mean values across the entire race. There was a significant linear trend toward a decrease (0.089%/km) in the mean difference in relative HR between the groups from start to finish. The amateurs’ HR relative to that of the elite group during the race could be described with the following linear regression:

Amateurs HR relative to elite HR (*d*) = 101.3 − 0.08866 × *d*, *R*^2^ = 0.69, *P* < 0.001, where *d* is the race distance in kilometers.

The regression models for the relative HR for the elite and amateur skiers’ were:

Elite:%HR_max_ (*d*) = 85.77 − 0.03995 × *d*, *R*^2^ = 0.062, *P* < 0.001
Amateurs:%HR_max_ (*d*) = 87.08 − 0.1286 × *d*, *R*^2^ = 0.41, *P* < 0.001

### Grip Wax Versus Exclusive DP

**FIGURE 5:**
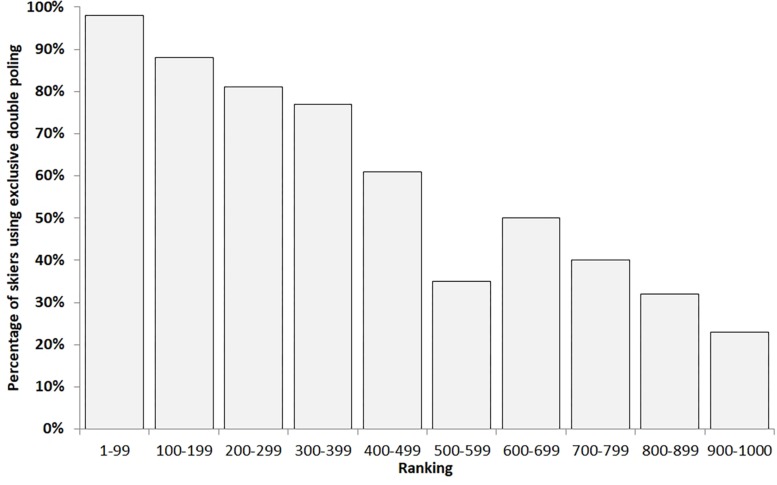
Percentage of skiers that used exclusive double poling (DP) during the first uphill (also assuming exclusive DP for the entire race) within performance groups in steps of 100 as based on their position during the uphill.

In the first climb, within the top 100 skiers, 98 athletes exclusively used the DP technique, with only two athletes employing the diagonal stride technique (e.g. using grip-waxed skis). As shown in Figure 5, the amount of skiers that exclusively used the DP technique almost steadily decreased toward the skiers ranked 900–1,000. Among the first 1,000 skiers, 59% applied exclusively the DP technique. Similar findings apply for the questionnaires where only four out of the included 19 athletes (21%) have used grip-waxed skis, with none in the elite group. Ten athletes (five in elite and five in amateur group) reported to have used specially designed DP skis.

## Discussion

The main findings of the current study were as follows: (1) elite skiers skied ∼15% faster but at equal relative exercise intensity across the 90-km Vasaloppet race as compared with amateur skiers (∼82% HR_max_); (2) while elite skiers were faster compared with amateur skiers in all five sections of the race, the greatest difference was found in section 4 (hilly and variable terrain at km 65–80) and lowest in sections 2 and 3 (sections including long downhills); (3) variability in skiing speeds within the groups was greatest in amateurs in section 1 (∼11% CV) and was progressively increasing from first to final section in elite (2% up to 9%); (4) elite skiers skied at a higher percentage in the high-intensity zones (zones 4 and 5) compared with amateur skiers (46 vs. 24%); they demonstrated a more evenly distributed pacing strategy, while amateurs demonstrated a more pronounced positive pacing (highest exercise intensity in the first section); the difference in exercise intensity between the two performance groups increased linearly across the 90-km race; (5) among the top 1,000 skiers, 59% exclusively used the DP technique, while this ratio decreased from the top 100 ranked in the first uphill (98%) down to 23% in the skiers ranked 901–1000; within the analyzed skiers, none of the elite used grip-waxed skis, while only four of the amateurs did; and (6) the by skiers applying GNSS HR monitors provide quite good agreement in horizontal distance (bias, 0.21%; limit of agreement of 0.90%) with quite high discrepancy in the total climb (bias, -17% with a limit of agreement of 30%).

### Exercise Intensity During the 90-km Vasaloppet

In the current study, it was demonstrated that elite and amateurs clearly differed with respect to skiing speed but competed on comparable mean relative HR across the entire 90-km race. The mean race exercise intensity was ∼82% HR_max_, with peak values of 96% HR_max_ being detected in the first long uphill at the start in 17 skiers and in the final spurt in the two fastest skiers. While the mean race HR is clearly lower as compared with studies over 5–15 km (mean >90% of HR_max_ and peak values of >96%) (Welde et al., 2003; Formenti et al., 2015; Stöggl et al., 2019), the peak values are on a comparable level, and for this race, distance and duration are quite high. In other sports, a clear trend toward reduced exercise intensity in relation to increased race duration can be observed. Neumayr et al. (2004) reported mean HR values of 68% during an ultramarathon cycling race in elite (27 h) and values of 77% during a 10-h race (Ötztal Radmarthon) in recreational cyclists (Neumayr et al., 2002). Analysis of fast and slow runners over distances of 10 km (fast, ∼33 min; slow, ∼40 min) and 21 km (fast, ∼74 min; slow, ∼94 min) revealed mean HR values of 89–91% of HR_max_ for both distances and performance groups (Selley et al., 1995). When plotting the results of the above-mentioned studies in different sports with respect to race duration (in h) versus mean race HR, an almost linear trend [HR_mean_ (bpm) = −0.8548 × race duration (h) + 89.27, *R*^2^ = 0.91] toward a reduction of 0.85%/h can be observed.

### Exercise Intensity Distribution During the 90-km Vasaloppet

In the current study, almost 85% of the race time was skied within the zones 2–4, while amateur skiers had a distinctly greater proportion in zone 2 compared with elite skiers (39 vs. 23%) but, in contrast, a clearly lower amount of race time in the high-intensity zones (zones 4 and 5: 24 vs. 46%). Although the mean race intensity was similar between both performance groups (∼82%), it was demonstrated that elite skiers were able to achieve a greater proportion in the high-intensity zones compared with the slower ones. To our knowledge, there is only the study of Formenti et al. (2015) reporting the exercise intensity distribution during XCS racing. In their 10-km race (mean race time, 25 min, 47 s), the proportion of HR being >90% (∼ zones 4 and 5 in the current study) was 67%; the remaining 23% was in the 80–90% HR_max_ zone (∼ zones 3 and 4), with no skiing performed in the low-intensity zones. In cycling, Neumayr et al. (2004) reported that, during a cycle ultramarathon (27 h), 53% of total race time was <70% HR_max_, 25% between 70 and 80%, 19% between 80 and 90%, and only 3% within the highest intensity zone (>90%). For recreational cyclists in the 10-h Ötztal marathon (Neumayr et al., 2002), a slight shift toward higher intensities was found being almost comparable with the intensity distribution of the elite skiers in the current study (18.5%, <70% HR_max_; 28%, 70–80% HR_max_; 39.5%, 80–90% HR_max_; and 14%, >90% HR_max_). Again, this result demonstrates that the data of the current study fit well into the pattern of a shift in intensity zones toward lower intensities the longer the duration of the race performed. However, it was also demonstrated that elite skiers seem to be able to achieve an intensity distribution toward higher intensities, while the amateur skiers toward lower intensity zones.

### Pacing Strategy Based on Heart Rate

Pacing strategy analysis revealed that the difference between the two performance groups was greater in the last two sections, compared with particularly sections 2 and 3. Therefore, we cannot confirm the finding of Carlsson et al. (2016), demonstrating that experienced skiers skied faster during the first half (positive pacing), whereas unexperienced skiers showed higher speed during the second half (negative pacing). Based on the low response rate of female skiers in the current study, no biological sex comparison was performed. With this regard, Nikolaidis and Knechtle (2017, 2018a) found that, in both races, men demonstrated more even pacing than women, in contrast to more even pacing in women compared with men in the study by Carlsson et al. (2016).

With respect to physiological loading, it was demonstrated that elite skiers clearly demonstrated a more even pacing strategy (decrease in HR of 0.04%/km), while amateur skiers showed distinct positive pacing (decrease in HR of 0.13%/km). The longer the completed total race distance, the greater the difference in HR between the two performance groups, with the difference between groups increasing linearly with 0.089%/km). Furthermore, the elite skiers were able to maintain a higher contribution in the high-intensity zones up to the end of the race, with two elite skiers even being able to reach their peak HR at the end of the race. For all other analyzed skiers, this was the case during the first long uphill of the race.

In the current study, the elite skiers demonstrated a more even pacing strategy both based on skiing speeds and HR response compared with the amateurs. This is partly in line with the study of Bilodeau et al. (1996) showing that during the 50-km classical race, the two best groups maintained a relatively consistent pace (i.e. even pacing), with the two worst groups being slower on the second half of the course than on the first half (i.e. positive pacing). However, with respect to the physiological response based on HR during the laps, no marked changes were observed.

In contrast to the HR response during the 90-km Vasaloppet, races over shorter distances generally reveal an increase in HR over race distance as previously observed by Stöggl et al. (2019), showing an increased mean and peak HR across two laps of a 5-km race simulation. Moreover, Formenti et al. (2015) showed a 2.42% increase in HR from the first to fourth lap of a 10-km race, while speed decreased across laps. Even in a 100-km cycling time trial of 2.5 h, an increase in HR of ∼8% was observed (St Clair Gibson et al., 2001). This increase in HR was proposed to reflect cardiovascular drift, resulting in a reduction in stroke volume (Coyle and Gonzalez-Alonso, 2001). In the running study of Selley et al. (1995), a constant running speed and HR response was demonstrated in both the 10- and 21-km race for both the fast and slow groups. Neumayr et al. (2004) reported a decline of 23% in exercise intensity for all participants during the race (86% during the first 6 h; down to 66% HR_max_ at the final period of the race). Therefore, in contrast to the above-mentioned phenomena of cardiac drift, a substantial decrease (10% every 10 h) in the HR response seems a general cardiovascular feature of ultramarathon cycling (Neumayr et al., 2004). HR was shown to decline at an average of 1–2%/h during cycling and triathlon events lasting 6–25 h, in both recreational (Neumayr et al., 2002; Laursen et al., 2005) and elite (Neumayr et al., 2004) athletes. This is emphasized by O’Toole et al. (1998) during prolonged running or cycling (>6 h), demonstrating a 6–7% decrease in exercise intensity based on HR. Compared to the current study, these previous results are comparable with the decline in HR for the amateur group, but are slightly divergent as compared to the elite skiers.

This decrease in HR during prolonged endurance competitions is a feature influenced by several factors such as substrate depletion (e.g. glycogen), altered substrate utilization, fluid and electrolyte imbalances, altered muscle efficiency, thermoregulatory problems, cardiac and neuromuscular fatigue, and psychological factors (O’Toole and Douglas, 1995; Neumayr et al., 2004). Furthermore, it was demonstrated that the lower the aerobic capacity, the greater the decline in performance across a race (Nikolaidis and Knechtle, 2018b). A high aerobic work capacity can assist athletes to maintain their race speed by preventing fatigue (Sjodin and Svedenhag, 1985; Coyle, 1995) based on higher VO_2__max_, anaerobic threshold performance, and running economy. Therefore, the ability of the current studies elite skiers to keep up a higher pace with less decline in physiological response across the 90-km race is most likely linked to the greater endurance capacity in sustaining higher power output throughout the entire race (Bilodeau et al., 1996). Furthermore, the slower, less experienced skiers may have chosen a poorer race strategy, with a fast initial pace (high proportion of skiing in the higher intensity zones), with a greater anaerobic contribution leading to earlier fatigue. As already pointed out by Bergstrom et al., 1973, after the Vasaloppet race, sufficient glycogen was being left in the legs, while arm glycogen stores were practically completely exhausted. In combination with the recently documented impressive increase in the upper body and DP capacity of modern XCS [e.g. improvement of peak aerobic work capacity with upper body exercises or DP in relation to whole-body exercise (e.g. running or diagonal stride) from 60 up to 95% in modern skiers] (Stöggl et al., 2019), it can be assumed that elite skiers have greater glycogen stores in the upper body or use a DP technique with a more advantageous energy supply (e.g. greater proportion of aerobic energy, especially based on fat) as compared with the amateur skiers.

The higher variability within section 1 in the amateur group might be an indication for a suboptimal pacing strategy in some of the amateur skiers (i.e. too high pace immediately after the start). For the elite skiers, the variability within the group grew steadily from section to section. Therefore, while the first parts of the race were based on tactics and drafting, and the low amount of breakaway attempts, a quite large group was able to stay together (unpublished data based on analysis of the entire race video). However, the longer the race, the more the discriminating factor within elite skiers. Most likely, those with less capacity, or using more demanding race tactics (e.g. skiing in front of the group, lead of breakaway attempts, filling gaps between groups, etc.) were no longer able to follow the lead group.

### Exclusive DP

The current study demonstrates that the trend toward exclusive application of the DP technique during a race seems to develop toward a mass phenomenon in long-distance XCS. In particular, 59% of the top 1,000 skiers in the first uphill used exclusively the DP technique, and even 23% of the 901–1,000 ranked skiers did so. The already mentioned importance of a well-developed upper body capacity during XCS is also fostered when using the strategy to exclusively DP a long-distance race. During the past three decades, in XCS, the DP technique has become more and more extensively utilized, and today, its successful usage determines the outcome of classic style XCS races (Holmberg et al., 2005; Stöggl and Holmberg, 2011; Sandbakk and Holmberg, 2014; Welde et al., 2017; Jonsson et al., 2019; Stöggl et al., 2019). Stöggl et al. (2019) recently demonstrated that elite male skiers were faster with exclusive DP compared with grip-waxed skis on an FIS-homologated race track over 5 km, but with no performance benefits for junior skiers and clearly lower in female juniors and seniors. This again strengthens the importance of a well-developed DP technique and upper body capacity to allow this strategy to work. To be mentioned here is that, in all races of the “Ski Classics” series within the last years, with the exception of the Reistadløpet, the winner (both men and women) uses exclusive DP. However, the Vasaloppet track records for both men and women are far faster with grip wax as compared to the records with exclusive DP (men: 3:38:41 in 2012 with grip wax vs. 3:57:18 with DP in 2017; women: 4:08:24 in 2012 vs. 4:17:56 in 2016) (Stöggl et al., 2019). The effects of the FIS “no-DP-zones” regulations needs further evaluation on how long and steep these sections need to be to produce sufficient negative impact of an imitated diagonal stride motion with no-grip wax to compensate the benefits of exclusive DP along the remaining parts of the track.

### Limitations and Perspectives

Based on the quite high amount of contacted athletes, coaches, and sport/ski clubs, the response rate can be seen as low (28 raw data files from the race were sent, while only 19 data sets were useful for analysis). Of note, only two female skiers replied to the questionnaire and sent their raw HR data. A great number of skiers reported that they did not use HR belts and monitors during the race. Finally, some skiers were not able to download their raw data from the software after uploading the data after the race (e.g. Strava). Another limitation can be seen in the HR_max_ data provided by the participants, which were not standardized and controlled. However, the race data with respect to relative HR response across the group seemed plausible. Although for long-distance races, HR is a good estimate of exercise intensity, it has some limitations like cardiovascular drift, dehydration, etc., and it does not cover intensities above VO_2__max_ (i.e. you miss out the supramaximal part). The video data of the first 1,000 ranked skiers in the first uphill do not necessarily mean that these skiers were also ranked top 1,000 at the end of the race. Furthermore, there was the assumption that if someone is using the DP technique in the analyzed video section, no grip wax was used. In some cases, it might have also been that the grip wax did not work (i.e. too slippery skis), forcing the skier to switch to DP.

## Conclusion and Practical Applications

The findings of the current study provides novel data about pacing strategies and physiological loads during a long-distance XCS skiing race for elite and amateur skiers. These data can be applied for recommendations regarding pacing strategy and intensity control during an XCS long-distance race. Too much skiing in a “high-intensity zone” (e.g. >90% HR_max_) in combination with inadequate timing (e.g. too high intensity in the first section of the race) might be problematic for the later phases within the race. This aspect can even be more severe when choosing the strategy of pure DP in non-elite skiers with assumed less trained upper-body capacities and therefore faster depletion of glycogen stores in the arms. While elite skiers demonstrated similar mean exercise intensities across the entire race compared with amateur skiers, they were clearly able to achieve more even pacing in general and particularly with respect to the high-intensity zone through the race. The majority of amateur skiers was shown to pace at a too high intensity at the beginning of the race, leading to a continuous decrease in exercise intensity across the race (distinct positive pacing both for speed and physiological response). This difference in the pacing profiles between these two groups demonstrate the greater capacity of elite skiers (aerobic capacity, energy stores, upper-body, and DP capacity), enhanced technical (more developed and economical technique) and tactical skills (avoiding too much skiing in high-intensity zones, drafting, timing of breakaway attempts), and possible more advantageous development of ski glide across the race compared with amateur or recreational skiers. The development of the strategy to exclusive apply DP across a broad range of performance levels (e.g. control sections for not skating), the importance of training of upper-body capacity, and to think about strategies for regulations to preserve all classical techniques are a future challenge for the race organizers.

## Data Availability Statement

The datasets generated for this study are available on request to the corresponding author.

## Ethics Statement

The studies involving human participants were reviewed and approved by the Ethics Committee, University of Salzburg. The patients/participants provided their written informed consent to participate in this study.

## Author Contributions

TS, BW, and MS conceptualized and designed the experiments. TS, EA, MS, and BW performed the experiments. TS, MH, and RB performed the data analysis. TS, MH, BW, MS, EA, and RB prepared the manuscript. All authors read and approved the final manuscript.

## Conflict of Interest

The authors declare that the research was conducted in the absence of any commercial or financial relationships that could be construed as a potential conflict of interest.

## Footnotes

1 www.vasaloppet.se
